# Age-stratified effects of intravenous ferric derisomaltose in heart failure with iron deficiency: insights from the IRONMAN trial

**DOI:** 10.1136/heartjnl-2024-324908

**Published:** 2025-02-12

**Authors:** Shirley Sze, Iain Squire, Paul R Kalra, John G Cleland, Mark C Petrie, Philip A Kalra, Fozia Ahmed, Prithwish Banerjee, Christopher J Boos, Callum Chapman, Peter James Cowburn, Lana Dixon, Simon Duckett, Rebecca Lane, Paul Foley, Ninian N Lang, Kristopher Lyons, Robin Ray, Rebekah Schiff, Elizabeth A Thomson, Michele Robertson, Ian Ford, IRONMAN Study group, Paul Kalra

**Affiliations:** 1 Department of Cardiovascular Sciences, University of Leicester, Leicester, UK; 2 Department of Cardiology, Portsmouth Hospitals University NHS Trust, Portsmouth, UK; 3 School of Cardiovascular and Metabolic Health, University of Glasgow, Glasgow, UK; 4 Department of Renal Medicine, Salford Royal Hospitals NHS Trust, Salford, UK; 5 Manchester Heart Centre, Manchester University NHS Foundation Trust, Manchester, UK; 6 Keele Cardiovascular Research Group, Keele University, Keele, UK; 7 Department of Cardiology, University Hospitals Coventry & Warwickshire, Coventry, UK; 8 Department of Cardiology, University Hospitals Dorset, Dorset, UK; 9 Chelsea and Westminster Hospital NHS Foundation Trust, London, UK; 10 Department of Cardiology, University Hospital Southampton, Southampton, UK; 11 Royal Victoria Hospital, Belfast, UK; 12 Department of Cardiology, University Hospital North Midlands, Stoke-on-Trent, UK; 13 Royal Brompton and Harefield Hospital, London, UK; 14 Great Western Hospitals NHS Foundation Trust, Swindon, UK; 15 Antrim Area Hospital, Antrim, UK; 16 St George's University Hospitals NHS Foundation Trust, London, UK; 17 Guy's and St Thomas' Hospitals NHS Trust, London, UK; 18 Robertson Centre for Biostatistics, University of Glasgow, Glasgow, UK

**Keywords:** heart failure

## Abstract

**Background:**

Intravenous iron therapy with ferric derisomaltose (FDI) has been shown to improve outcomes in patients with heart failure with reduced ejection fraction (HFrEF) and iron deficiency. However, its effects across different age groups remain unclear. This analysis of the Effectiveness of Intravenous Iron Treatment versus Standard Care in Patients with Heart Failure and Iron Deficiency (IRONMAN) trial explored the efficacy and safety of FDI across age groups.

**Methods:**

The IRONMAN trial was a prospective, open-label, blinded end point randomised controlled trial enrolling patients with HFrEF and iron deficiency. This prespecified analysis stratified the population into four quarters by age group: <67 years, 67–73 years, 74–79 years, >79 years. The primary outcome was a composite of recurrent heart failure hospitalisations and cardiovascular death. Secondary outcomes included changes in haemoglobin and quality of life. Clinical outcomes comparing FDI versus usual care in each age subgroup were analysed by the method of Lin *et al* for recurrent events and Cox proportional hazards model for time to first event. Interactions between age and treatment effects were explored.

**Results:**

Among 1137 randomised patients (median age 73 years), the primary outcome rate ratio (FDI vs usual care) was 0.87 (95% CI 0.61 to 1.23) in patients <67 years, 0.93 (95% CI 0.66 to 1.32) in those aged 67–73 years, 0.88 (95% CI 0.59 to 1.33) in those aged 74–79 years and 0.66 (95% CI 0.45 to 0.96) in those aged >79 years (p-interaction=0.38). Improvements in haemoglobin and quality of life scores at 4 months did not differ statistically across age groups (p-interaction=0.92 and 0.64, respectively). Older patients were more symptomatic at baseline, with higher N-terminal-pro B-type natriuretic peptide levels and poorer renal function, but safety outcomes did not differ across age groups.

**Conclusions:**

We found no evidence that the effects of FDI on heart failure hospitalisations, cardiovascular death, haemoglobin and quality of life differed by age. These findings support its use in patients with HFrEF and iron deficiency, including older adults.

**Trial registration number:**

NCT02642562.

**WHAT IS ALREADY KNOWN ON THIS TOPIC**

- Several randomised trials of patients with heart failure with reduced ejection fraction (HFrEF) and iron deficiency report that administration of intravenous iron improved symptoms and quality of life (QoL) and reduced clinical events.
- For older patients, high levels of comorbidity and frailty could either limit or enhance the benefits of correcting iron deficiency.

**WHAT THIS STUDY ADDS**

- Our analysis found no attenuation of the benefits of intravenous ferric derisomaltose on changes in haemoglobin or improvement in QoL or clinical outcomes in older compared with younger patients with HFrEF.

**HOW THIS STUDY MIGHT AFFECT RESEARCH, PRACTICE OR POLICY**

- IRONMAN enrolled older patients with HFrEF than in many previous trials of intravenous iron.
- A quarter of participants were aged >79 years, which is close to the mean age of patients with HFrEF encountered in clinical practice in the UK and other high-income countries.
- Older patients with HFrEF and iron deficiency benefit from intravenous iron.

## Introduction

 Iron deficiency is common in patients with heart failure (HF) and a marker of poor prognosis.^1^ Iron deficiency is associated with higher symptom burden, poorer exercise capacity, worse quality of life (QoL) and an increased risk of hospitalisation and death.^1^,^3^ The prevalence of iron deficiency also increases with age.^4^ While oral iron supplements are widely prescribed, their usefulness is greatly limited by poor absorption, gastrointestinal side effects and poor compliance. Moreover, oral iron supplementation does not improve exercise capacity or QoL in patients with left ventricular ejection fraction (LVEF) <40%.^5^ Several randomised controlled trials^6^,^9^ have demonstrated that treatment of iron deficiency with intravenous ferric derisomaltose (FDI) and ferric carboxymaltose is associated with an improvement in symptoms and QoL in patients with HFrEF and might reduce hospitalisation for HF. However, in older people with HF, the tolerability and benefits of intravenous iron is uncertain.^8^

In the Effectiveness of Intravenous Iron Treatment versus Standard Care in Patients with Heart Failure and Iron Deficiency (IRONMAN) trial (NCT02642562), FDI in patients with LVEF ≤45% (hereafter referred to as HF), resulted in a borderline significant reduction in HF hospitalisations and cardiovascular (CV) deaths (statistically significant in the prespecified COVID-19 sensitivity analysis), as well as improvement in QoL at 4 months, compared with usual care (UC).^9^ To aid shared decision-making, it is important to understand the efficacy and safety of FDI in patients with HFrEF in different age groups. IRONMAN enrolled older patients than many previous trials of intravenous iron in HF, with a mean age of 73 years and a quarter of participants over 79 years of age, closely resembling contemporary HF populations encountered in routine clinical practice. Here, we explore the influence of age on treatment outcomes, QoL and change in haemoglobin (Hb) concentration in the IRONMAN trial.

## Methods

### IRONMAN trial design

The design and primary results of IRONMAN, a UK-based, multicentre, investigator-initiated, prospective, open-label, blinded end point, event-driven randomised controlled trial, have been published previously.^9 10^

### Patient population

IRONMAN included patients aged ≥18 years with new or established symptomatic HF (current or recent (within 6 months) HF hospitalisation; or outpatient with raised plasma concentration of natriuretic peptides (N-terminal-pro B-type natriuretic peptide (NT-proBNP) >250 ng/L or BNP >75 ng/L in sinus rhythm; or NT-proBNP >1000 ng/L or BNP >300 ng/L in atrial fibrillation), evidence of iron deficiency (serum ferritin <100 µg/L or transferrin saturation (TSAT) <20%) and an LVEF ≤45% in the preceding 24 months. The study excluded patients with serum ferritin concentrations >400 µg/L, Hb concentration <90 g/L or >140 g/L in men or >130 g/L in women or an estimated glomerular filtration rate <15 mL/min/1.73 m^2^.

### Treatment arms

Patients were randomised (1:1) to receive FDI and UC or UC alone, stratified by recruitment context and trial site. FDI was given open-label with blinded adjudication of outcomes. Patients were reviewed 4 weeks postrandomisation, followed by 4-monthly visits until trial completion. Patients randomised to FDI were given FDI, administered via infusion at a dose determined by body weight and Hb concentration as previously described.^10^ In the UC group, patients were allowed oral iron at the discretion of the responsible clinicians, although this was not actively encouraged. In both groups, investigators were encouraged to optimise HF therapy according to guidelines.^11^

### Study end points and assessments

The primary end point was a composite of total HF hospitalisations and CV death (recurrent events analysis). Secondary end points included CV hospitalisations; CV death; all-cause death and all-cause hospitalisations. Other end points included QoL at 4 months (assessed using the Minnesota Living with Heart Failure Questionnaire (MLHFQ), including overall, physical and emotional scores) and change in serum Hb from baseline to 4 months.

The MLHFQ is a self-administered disease-specific QoL questionnaire for patients with HF.^12^ It has 21 items rated on 6-point Likert scales, representing various degrees of impact of HF on QoL, from 0 (none) to 5 (very much). The total score ranges from 0 (best QoL) to 105 (worse QoL). Apart from the total score, MLHFQ also scores for two dimensions: physical (eight items, range 0–40) and emotional (five items, range 0–25). We evaluated MLHFQ (total score, physical and emotional dimensions) rather than EQ5D and 6 min walk test distance as these outcomes were similar at 4 months between treatment groups in the main study. We did not report on change in MLHFQ from baseline to 4 months as some patients were recruited in hospital and some were recently discharged from hospital, for whom MLHFQ may not be meaningful. Deaths and hospitalisations due to infection were prespecified safety outcomes.

### Statistical methods

Patients were analysed in quarters of the distribution of age (Q1: age <67 years; Q2: age ≥67–≤73 years; Q3: >73–≤79 years and Q4: >79 years). Baseline characteristics were summarised as median (IQR) for continuous variables and n (%) for categorical variables and compared across age subgroups using Kruskal-Wallis and χ^2^ tests as appropriate.

All analyses were applied in the validly randomised population. Primary and secondary clinical outcomes comparing FDI versus UC within each subgroup were analysed by the method of Lin *et al* for recurrent events (treatment effect estimated in the form of event rate ratios (RRs) and 95% CIs) and Cox proportional hazards model for time-to-first-event outcomes (treatment effect presented as HRs and 95% CI). Interaction p values evaluating the interaction between age and the effect of FDI versus UC on clinical outcomes were reported.

In each age subgroup, mean (SD) MLHFQ (overall, physical and emotional scores) at 4 months and mean (SD) change in serum Hb (4 months minus baseline) were compared between treatment groups using analysis of co-variance. 95% CIs for treatment effects were reported for each age subgroup and p values for the interaction between age and the effect of FDI were reported. Infection-related deaths and hospitalisations were analysed as time to first events.

All analyses used R V.4.3.2 or Minitab V.20.4.2. P values <0.05 were considered statistically significant.

## Results

### Baseline characteristics by age

Baseline characteristics of the 1137 validly randomised patients are shown by quarters of age in table 1. The median duration of follow-up was 2.7 (IQR 1.8-3.6) years. Older patients were more symptomatic, had higher serum NT-proBNP and worse renal function compared with younger patients. They also had higher burden of comorbidity, such as atrial fibrillation and hypertension. There was no difference in Hb, serum ferritin or TSAT among the age groups.

**Table 1:** Baseline characteristics by quarters of age

	Age groups (years)				P value
	<67(n=285)	≥67–≤73 (n=284)	>73–≤79 (n=283)	>79(n=285)	
Age (years)	60 (55, 64)	72 (67, 72)	76 (75, 78)	83 (81, 86)	–
Women	85 (30)	84 (30)	62 (22)	69 (24)	0.077
White	242 (85)	265 (93)	262 (93)	274 (96)	<0.001
Recruitment context					0.93
In hospital	38 (13)	42 (15)	39 (14)	45 (16)	
Recent discharge	58 (21)	49 (17)	49 (17)	52 (18)	
Ambulatory patient	189 (66)	193 (68)	195 (69)	188 (66)	
Systolic BP (mm Hg)	114 (103, 130)	118 (106, 132)	122 (109, 133)	120 (108, 132)	0.003
LVEF (%)	30 (25, 35)	35 (27, 38)	35 (28, 38)	34 (25, 39)	0.001
BMI	29.0(24.9–33.5)	29.7(25.9–33.8)	28.1(24.6–32.3)	27.0(23.6–29.9)	<0.001
NYHA III/IV	107 (38)	120 (42)	121 (43%)	141 (49)	0.038
Ischaemic aetiology	154 (54)	168 (59)	159 (56)	166 (58)	0.61
Atrial fibrillation	85 (30)	132 (46)	153 (54)	164 (58)	<0.001
Hypertension	120 (42)	159 (56)	170 (60)	163 (57)	<0.001
Diabetes	139 (49)	143 (50)	137 (48)	102 (36)	0.001
Hb (g/dL)	12.3 (11.2, 12.9)	12.1 (11.3, 12.9)	12.1 (11.2, 12.8)	11.8 (11.1, 12.7)	0.079
TSAT (%)	15 (10, 19)	15 (11, 20)	16 (11, 19)	16 (11,21)	0.32
TSAT <20%	221 (79)	207 (75)	208 (76)	205 (73)	0.44
Ferritin (ng/mL)	54 (28, 90)	48 (28, 82)	48 (31, 85)	49 (30, 84)	0.77
eGFR (mL/min/1.73 m^2^)	64 (48, 86)	54 (39, 71)	47 (37, 73)	44 (34, 55)	<0.001
NT-proBNP (ng/L)*	1203 (607, 3023)	1414 (820, 2823)	1686 (964, 3602)	2745 (1570, 4822)	<0.001

Continuous variables are summarised as median (lower quartile, upper quartile) and categorical variables as counts and percentages.

* NT-proBNP was recorded only in a subset of ambulatory patients: 142, 136, 144 and 143 patients had NT-proBNP recorded for age groups <67 years, ≥67–≤73 years, >73–≤79 years and >79 years, respectively.

BMI, body mass index; BP, blood pressure; eGFR, estimated glomerular filtration rate; FDI, intravenous ferric derisomaltose; Hb, haemoglobin; LVEF, left ventricular ejection fraction; NT-proBNP, N-terminal-pro B-type natriuretic peptide; NYHA, New York Heart Association; TSAT, transferrin saturation.

### Treatment effect by age

**Figure 1:**
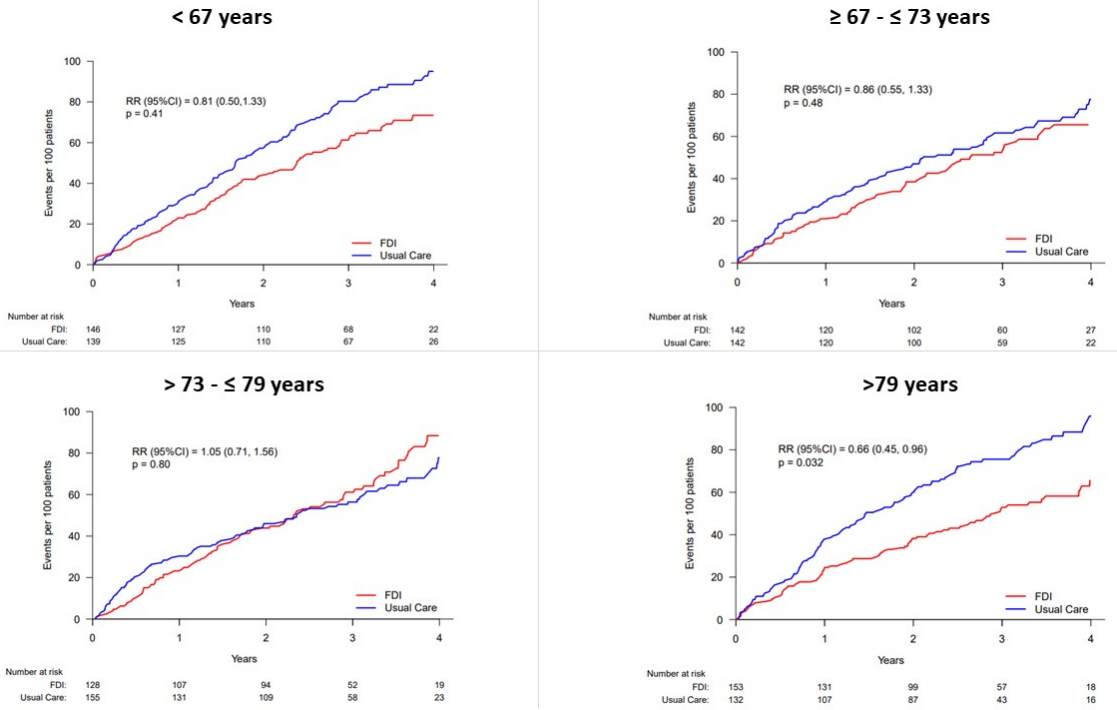
Rates of recurrent heart failure hospitalisations and cardiovascular death by age group. FDI, intravenous ferric derisomaltose; RR, rate ratio.

The event rates (events/100 patient-years) for the primary outcome were nominally lower in the FDI arm compared with UC across all age quarters, other than Q3 (>73–≤79 years). In Q4 (>79 years), the event rates for the primary outcome was statistically lower in the FDI arm compared with UC (20.6 vs 33.1), with an RR (95% CI) of 0.66 (0.45, 0.96), p=0.032 (figure 1; table 2). Although the absolute difference in RR of FDI relative to UC for the primary outcome was numerically greatest in the oldest quarter of age, there was no evidence of interaction between age and treatment effect (p-interaction=0.38) (table 2). A similar pattern of both primary outcome event rates and the treatment effect of FDI versus UC was similar when the analysis was restricted to patients with TSAT <20% (table 2). The treatment effect of FDI versus UC was similar for the primary outcome assessed using a time-to-first-event analysis, and for secondary outcomes including CV death, all-cause death and all-cause hospitalisations (table 2).

**Table 2:** Primary, secondary and infection-related end points according to quarters of age

	Age groups (years)								P interaction
	<67(n=285)		≥67–≤73(n=284)		>73–≤79(n=283)		>79(n=285)		
	FDI(n=146)	UC(n=139)	FDI(n=142)	UC(n=142)	FDI(n=128)	UC(n=155)	FDI(n=153)	UC(n=132)	
CV death and HF hospitalisations (recurrent)									
N, rate/100 patient-years	91 (22.8)	113 (28.1)	78 (20.6)	94 (24.9)	88 (26.2)	99 (24.8)	79 (20.6)	105 (33.1)	
HF hospitalisation events	64	87	52	62	59	62	42	62	
Rate ratio (95% CI)	0.81 (0.50, 1.33)		0.86 (0.55, 1.33)		1.05 (0.71, 1.56)		0.66 (0.45, 0.96)		0.38
CV death and HF hospitalisations (recurrent)—TSAT <20%									
N, rate/100 patient-years	67 (22.4)N=112	105 (33.4)N=109	58 (23.0)N=98	69 (23.7)N=109	67 (26.5)N=94	71 (26.2)N=114	62 (23.1)N=109	78(34.6)N=96	
Rate ratio (95% CI)	0.66 (0.39, 1.11)		0.97 (0.59, 1.58)		1.00 (0.63, 1.59)		0.70 (0.45, 1.11)		0.45
CV death and HF hospitalisations (first)									
N (%)	47 (32)	52 (37)	46 (32)	56 (39)	50 (39)	64 (41)	55 (36)	59 (45)	
HR (95% CI)	0.85 (0.57, 1.27)		0.88 (0.59, 1.30)		0.90 (0.62, 1.31)		0.80 (0.55, 1.15)		0.96
CV death									
N (%)	27 (18)	26 (19)	26 (18)	32(23)	29 (23)	37 (24)	37 (24)	43 (33)	
HR (95% CI)	0.97 (0.56, 1.68)		0.82 (0.49, 1.38)		0.92 (0.56, 1.49)		0.72 (0.46, 1.12)		0.80
All-cause death									
N (%)	39 (27)	38 (27)	41 (29)	45 (32)	43 (34)	50 (32)	61 (40)	60 (45)	
HR (95% CI)	1.01 (0.64, 1.58)		0.93 (0.61, 1.42)		1.01 (0.67, 1.52)		0.85 (0.59, 1.22)		0.90
All-cause hospitalisation									
N (%)	85 (58)	78 (56)	82 (58)	97 (68)	87 (68)	108 (70)	97 (63)	87 (66)	
HR (95% CI)	1.08 (0.79, 1.48)		0.83 (0.62, 1.12)		0.91 (0.68, 1.20)		0.88 (0.66, 1.18)		0.65
Fatal infection									
N (%)	4 (3)	7 (5)	7 (5)	7 (5)	8 (6)	5 (3)	15 (10)	9 (7)	
HR (95% CI)	0.65 (0.19, 2.23)		1.01 (0.35, 2.89)		1.96 (0.64, 6.00)		1.37 (0.60, 3.16)		0.53
Infection-related hospitalisation									
N (%)	23 (16)	30 (22)	30 (21)	43 (30)	25 (20)	39 (25)	33 (22)	28 (21)	
HR (95% CI)	0.74 (0.43, 1.29)		0.64 (0.40, 1.03)		0.69 (0.42, 1.14)		1.01 (0.61, 1.68)		0.66

CV, cardiovascular; FDI, intravenous ferric derisomaltose; HF, heart failure; MI, myocardial infarction; UC, usual care.

### Change in Hb

**Figure 2:**
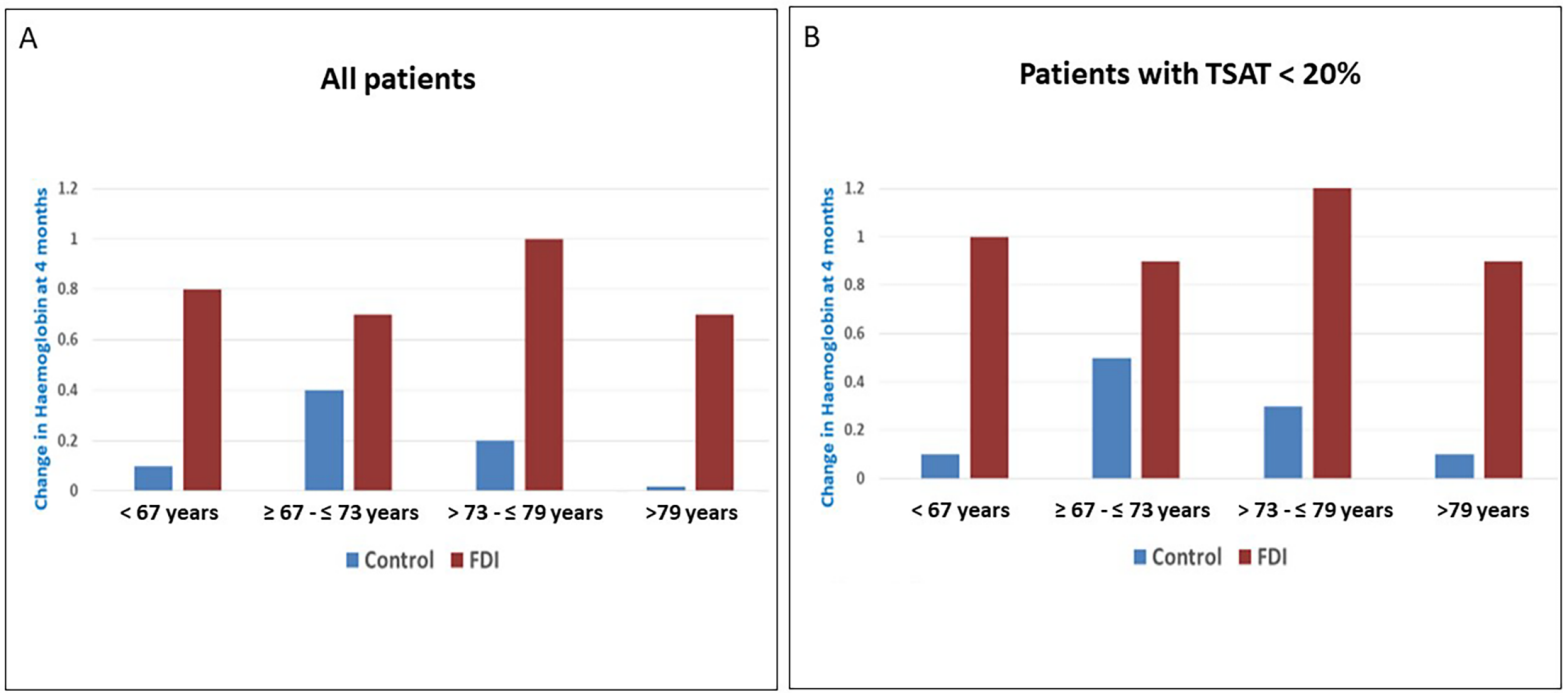
Change in haemoglobin levels at 4 months across age groups. FDI, intravenous ferric derisomaltose; TSAT, transferrin saturation.

Hb concentrations at 4 months were higher in the FDI arm compared with UC across all age groups (table 3). There was no evidence of any difference in the effect of FDI on change in Hb concentration (4 months minus baseline) according to age (P interaction=0.17) (table 3). Observations were similar when the analysis was restricted to patients with TSAT <20% (figure 2).

**Table 3:** Change in haemoglobin (baseline vs 4 months) and MLHFQ at 4 months according to quarters of age

	Age groups (years)												P interaction
	<67(n=284)			≥67–≤73(n=284)			>73–≤79(n=283)			>79(n=285)			
	FDI(n=146)	UC(n=138)	Treatment effect(95% CI, p value)	FDI(n=142)	UC(n=142)	Treatment effect(95% CI, p value)	FDI(n=128)	UC(n=155)	Treatment effect(95% CI, p value)	FDI(n=153)	UC(n=132)	Treatment effect(95% CI, p value)	
Haemoglobin (g/dL)													
BL	12.1(1.2)	12.0(1.2)		12.1(1.1)	12.0(1.2)		12.0(1.1)	12.0(1.2)		11.8(1.1)	11.9(1.0)		
4 m	13.0(1.4)N=113	12.1(1.6)N=113		12.9(1.3)N=109	12.4(1.4)N=114		12.9(1.3)N=101	12.3(1.4)N=110		12.5(1.4)N=127	12.0(1.3)N=101		
Δ (BL vs 4 m)	0.8(1.5)N=113	0.1(1.1)N=113	0.7(0.4, 1.1)<0.001	0.7(1.4)N=109	0.4(1.2)N=114	0.3(−0.2, 0.7)0.07	1.0(1.3)N=101	0.2(1.3)N=110	0.8(0.5, 1.2)<0.001	0.7(1.2)N=127	0.0(1.2)N=101	0.7(0.4, 1.0)<0.001	0.17
MLHFQ (4 m)													
Overall (4 m)	49(30)N=122	47(27)N=112	2(−6, 9)0.70	36(28)N=128	39(27)N=127	−3(−10, 4)0.35	32(25)N=114	40(26)N=127	−8(−15, –2)0.014	32(24)N=138	36(25)N=118	−4(−10, 2)0.21	0.26
Physical (4 m)	22(12)N=121	21(12)N=112	1(−3, 4)0.78	18(12)N=128	20(12)N=127	–3(−6, 0)0.094	16(11)N=112	20(11)N=127	–4(−7, –1)0.007	17(11)N=138	19(11)N=117	–2(−5, 1)0.14	0.23
Emotional (4 m)	12(9)N=122	12(8)N=113	0(−2, 2)0.95	8(8)N=128	9(8)N=127	0(−2, 2)0.90	7(7)N=114	8(7)N=127	–2(−4, 0)0.086	7(7)N=138	7(7)N=117	–1(−2, 1)0.61	0.64

BL, baseline; FDI, intravenous ferric derisomaltose; m, month; MLHFQ, Minnesota Living with Heart Failure Questionnaire; UC, usual care.

### Disease-specific QoL

MLHFQ (overall scores) at 4 months were numerically lower (better QoL) in the FDI arm across all age subgroups apart from Q1, where the scores were similar between treatment arms (table 3). The differences in overall scores were mainly driven by difference in MLHFQ physical scores. There were no statistically significant interactions between age and the effect of FDI versus UC on MLHFQ scores at 4 months (table 3).

### Infection-related deaths and hospitalisations

The numbers of infection-related deaths were too few to permit meaningful analysis (table 2). Rates of first infection-related hospitalisations were numerically lower for patients in the FDI arm, with no evidence of an interaction with age (table 2).

## Discussion

Patients enrolled in IRONMAN^9^ were older compared with those in many previous trials of intravenous iron in HF,^6^,^8^,^13^ with a mean age of 73 years and a quarter aged >79 years, closely resembling contemporary patients encountered in routine clinical practice.^14^ Our analysis shows no evidence of attenuation of the effects of FDI on major clinical outcomes, QoL or Hb concentrations in older patients.

As observed in previous trials,^15^ we found that older patients with HF and iron deficiency displayed features of more advanced HF, with higher NT-proBNP and greater symptom burden compared with younger patients. They also have a greater burden of comorbidity, for example, atrial fibrillation and chronic kidney disease, which may in part explain the higher NT-proBNP concentrations. Older patients are more likely to require anticoagulation for atrial fibrillation, which may further increase the risk of iron deficiency and anaemia. In this context, the observation of similar impact of FDI on prognosis, Hb and QoL irrespective of age is of clinical relevance.

Several studies have demonstrated beneficial effects of medical therapy in patients with HF across the age spectrum.^16 17^ Our study suggests that FDI can be added to contemporary medical therapy to improve outcomes in patients with HF and iron deficiency, irrespective of age. The lack of interaction between age and outcomes with intravenous iron in IRONMAN is in agreement with previous observations in a trial of ferric carboxymaltose compared with placebo, in patients hospitalised due to worsening HF and concomitant iron deficiency.^8^ Together, these studies suggest that intravenous iron is beneficial in patients with HF with iron deficiency, irrespective of age. Interestingly, the reduction in the primary outcome with FDI was greatest in absolute terms for patients in the oldest quarter of age (aged >79 years).

A recent meta-analysis (comprising IRONMAN and AFFIRM-AHF) evaluating the clinical efficacy of intravenous iron in patients with HF and iron deficiency concludes consistent efficacy across age groups.^18^ This should be considered in the context of important differences in the populations recruited to the two trials.^8 9^ IRONMAN enrolled mostly outpatients, whereas AFFIRM-AHF recruited solely patients during a hospital admission for HF. Patients in IRONMAN were at a lower risk of events but were also older than in AFFIRM-AHF. IRONMAN therefore extends our understanding of the benefits of intravenous iron to a much broader range of patients with HF. Follow-up was longer in IRONMAN (median follow-up 2.7 years) compared with AFFIRM-AHF, in which intravenous iron was not given after 24 weeks and follow-up stopped at 12 months. Therefore, IRONMAN provides important data on the longer-term safety of intravenous iron. These factors may explain why the benefits of FDI in the elderly were more clearly observed in IRONMAN compared with AFFIRM-AHF.

Furthermore, we found no evidence of interactions between age and the effect of FDI compared with UC on QoL at 4 months assessed using the MLHFQ, in agreement with previous observations in similar patient cohorts.^6^ This may be particularly relevant for older patients, for whom improvement in QoL may be the main aim of treatment.

In the primary outcome analysis, we did not observe a numerical superiority of FDI compared with UC in Q3 compared with the other quarters of age. In the QoL analysis, we did not observe any numerical improvement in QoL at 4 months in FDI versus UC arm in Q1 compared with the other quarters of age. Such observations may be due to subdivision of the study population, resulting in limited statistical power to demonstrate a difference in outcome between the two treatment groups.

In this analysis, the improvement in Hb concentration of FDI versus UC (baseline vs 4 months) was seen irrespective of age. This aligns with the well-established relationship among iron repletion, increased Hb concentration and improved QoL.^7^

FDI was not associated with an increase in infection-related hospitalisation in older patients; in fact, we observed that a lower proportion of patients had an infection-related hospitalisation in the FDI versus UC arm across the age spectrum. Interestingly, the rate of fatal infection was higher in older than younger patients in FDI versus UC arms, although the number of fatal infections was low. This is likely related to greater disease burden in older patients.

Our study has limitations, including low statistical power to identify an interaction between treatment and age. IRONMAN recruited predominantly Caucasian patients, and our observations may not generalise well to patients of other ethnicities. IRONMAN was an open-label study, theoretically influencing the reliability of subjective end points, such as QoL. The inclusion and exclusion criteria of IRONMAN might have restricted the enrolment of very high-risk patients. However, our cohort showed marked elevation of natriuretic peptide concentrations, had high burden of renal impairment and other comorbidity, and a quarter were aged >79 years, although we were unable to take into account the frailty status of patients in the analysis.

## Conclusion

In the IRONMAN trial, older patients with HFrEF and iron deficiency were more symptomatic with higher NT-proBNP and worse renal function. There was no evidence that the effects of FDI on HF hospitalisations, CV death, Hb and QoL differed by age. Our data support the use of intravenous iron therapy in older patients with HFrEF and iron deficiency.

## Data Availability

Data are available on reasonable request.

## References

[1] Cleland JGF, Zhang J, Pellicori P (2016). Prevalence and Outcomes of Anemia and Hematinic Deficiencies in Patients With Chronic Heart Failure. JAMA Cardiol.

[2] von Haehling S, Gremmler U, Krumm M (2017). Prevalence and clinical impact of iron deficiency and anaemia among outpatients with chronic heart failure: The PrEP Registry. Clin Res Cardiol.

[3] Enjuanes C, Klip IT, Bruguera J (2014). Iron deficiency and health-related quality of life in chronic heart failure: results from a multicenter European study. Int J Cardiol.

[4] Alnuwaysir RIS, Hoes MF, van Veldhuisen DJ (2021). Iron Deficiency in Heart Failure: Mechanisms and Pathophysiology. J Clin Med.

[5] Lewis GD, Malhotra R, Hernandez AF (2017). Effect of Oral Iron Repletion on Exercise Capacity in Patients With Heart Failure With Reduced Ejection Fraction and Iron Deficiency: The IRONOUT HF Randomized Clinical Trial. JAMA.

[6] Anker SD, Comin Colet J, Filippatos G (2009). Ferric carboxymaltose in patients with heart failure and iron deficiency. N Engl J Med.

[7] Ponikowski P, van Veldhuisen DJ, Comin-Colet J (2015). Beneficial effects of long-term intravenous iron therapy with ferric carboxymaltose in patients with symptomatic heart failure and iron deficiency†. Eur Heart J.

[8] Ponikowski P, Kirwan B-A, Anker SD (2020). Ferric carboxymaltose for iron deficiency at discharge after acute heart failure: a multicentre, double-blind, randomised, controlled trial. Lancet.

[9] Kalra PR, Cleland JGF, Petrie MC (2022). Intravenous ferric derisomaltose in patients with heart failure and iron deficiency in the UK (IRONMAN): an investigator-initiated, prospective, randomised, open-label, blinded-endpoint trial. Lancet.

[10] Kalra PR, Cleland JG, Petrie MC (2022). Rationale and design of a randomised trial of intravenous iron in patients with heart failure. Heart.

[11] McDonagh TA, Metra M, Adamo M (2021). 2021 ESC Guidelines for the diagnosis and treatment of acute and chronic heart failure. Eur Heart J.

[12] Rector TS, Kubo SH, Cohn JN (1987). Patients’ self-assessment of their congestive heart failure. Part 2: content, reliability and validity of a new measure, The Minnesota Living with Heart Failure Questionnaire. Heart Fail.

[13] Mentz RJ, Garg J, Rockhold FW (2023). Ferric Carboxymaltose in Heart Failure with Iron Deficiency. N Engl J Med.

[14] Veenis JF, Brunner-La Rocca H-P, Linssen GC (2019). Age differences in contemporary treatment of patients with chronic heart failure and reduced ejection fraction. Eur J Prev Cardiol.

[15] Savarese G, von Haehling S, Butler J (2023). Iron deficiency and cardiovascular disease. Eur Heart J.

[16] Martinez FA, Serenelli M, Nicolau JC (2020). Efficacy and Safety of Dapagliflozin in Heart Failure With Reduced Ejection Fraction According to Age: Insights From DAPA-HF. Circulation.

[17] Murphy SP, Ward JH, Piña IL (2022). Age Differences in Effects of Sacubitril/Valsartan on Cardiac Remodeling, Biomarkers, and Health Status. JACC Heart Fail.

[18] Graham FJ, Pellicori P, Kalra PR (2023). Intravenous iron in patients with heart failure and iron deficiency: an updated meta-analysis. Eur J Heart Fail.

